# Inhibition of Recruitment and Activation of Neutrophils by Pyridazinone-Scaffold-Based Compounds

**DOI:** 10.3390/ijms23137226

**Published:** 2022-06-29

**Authors:** Aurélie Moniot, Julien Braux, Renaud Siboni, Christine Guillaume, Sandra Audonnet, Ingrid Allart-Simon, Janos Sapi, Rabindra Tirouvanziam, Stéphane Gérard, Sophie C. Gangloff, Frédéric Velard

**Affiliations:** 1 Université de Reims Champagne-Ardenne, EA 4691 BIOS, 51 Rue Cognacq Jay, 51100 Reims, France; aurelie.moniot@univ-reims.fr (A.M.); julien.braux@univ-reims.fr (J.B.); renaud.siboni@univ-reims.fr (R.S.); christine.guillaume@univ-reims.fr (C.G.); sophie.gangloff@univ-reims.fr (S.C.G.); 2 Université de Reims Champagne-Ardenne, URCACyt, 51 Rue Cognacq Jay, 51100 Reims, France; sandra.audonnet@univ-reims.fr; 3 Université de Reims Champagne-Ardenne, UMR CNRS 7312 ICMR, 51 Rue Cognacq Jay, 51100 Reims, France; ingrid.allart-simon@univ-reims.fr (I.A.-S.); janos.sapi@univ-reims.fr (J.S.); stephane.gerard@univ-reims.fr (S.G.); 4 Department of Pediatrics, Emory University School of Medicine, Atlanta, GA 30322, USA; tirouvanziam@emory.edu; 5 Center for CF and Airways Disease Research, Children’s Healthcare of Atlanta, 2015 Uppergate Road, Atlanta, GA 30322, USA

**Keywords:** air pouch model, exocytosis, inflammation, migration, phagocytosis

## Abstract

In inflammatory diseases, polymorphonuclear neutrophils (PMNs) are known to produce elevated levels of pro-inflammatory cytokines and proteases. To limit ensuing exacerbated cell responses and tissue damage, novel therapeutic agents are sought. 4aa and 4ba, two pyridazinone-scaffold-based phosphodiesterase-IV inhibitors are compared in vitro to zardaverine for their ability to: (1) modulate production of pro-inflammatory mediators, reactive oxygen species (ROS), and phagocytosis; (2) modulate degranulation by PMNs after transepithelial lung migration. Compound 4ba and zardaverine were tested in vivo for their ability to limit tissue recruitment of PMNs in a murine air pouch model. In vitro treatment of lipopolysaccharide-stimulated PMNs with compounds 4aa and 4ba inhibited the release of interleukin-8, tumor necrosis factor-α, and matrix metalloproteinase-9. PMNs phagocytic ability, but not ROS production, was reduced following treatment. Using a lung inflammation model, we proved that PMNs transmigration led to reduced expression of the CD16 phagocytic receptor, which was significantly blunted after treatment with compound 4ba or zardaverine. Using the murine air pouch model, LPS-induced PMNs recruitment was significantly decreased upon addition of compound 4ba or zardaverine. Our data suggest that new pyridazinone derivatives have therapeutic potential in inflammatory diseases by limiting tissue recruitment and activation of PMNs.

## 1. Introduction

Polymorphonuclear neutrophils (PMNs) are crucial in the first line of defense against pathogens, as evidenced by the fact that their peripheral count increases in case of bacterial infection [1]. After transmigration from blood into infected tissues, PMN functions are activated. These include an increase in phagocytosis, reactive oxygen species (ROS) production, degranulation, and generation of neutrophil-extracellular traps (NETs) [2]. PMN phagocytosis requires complement receptors (CR1 (CD35) and CR3 (CD11b/CD18)) or Fcγ (FcγRIIA (CD32) and FcγRIIIB (CD16)) to enable pathogen uptake [3,4]. Notably, downregulation of surface CD16 has also been used as a marker of PMN apoptosis [5]. Once taken up, bacteria are enclosed inside phagosomes, where ROS create an extremely toxic, microbicidal environment [6]. To kill bacteria extracellularly, PMNs undergo gradual mobilization of vesicular and granular compartments, starting with secretory vesicles and tertiary granules (containing, among other effectors, the vesicle-associated membrane protein (VAMP) and matrix metalloproteinase-9 (MMP-9), respectively). A hallmark of this process is an increase in surface expression of the CD16 receptor on PMNs. Following tertiary granules, secondary granules are exocytosed. This is manifested by the extracellular release of lactoferrin and an increase in surface expression of CD66b. Lastly, the exocytosis of primary granules containing anti-microbial proteins and neutrophil elastase (NE) may ensue, which is marked by an increase in surface expression of CD63 [7,8]. In chronic obstructive pulmonary disease (COPD) or cystic fibrosis (CF), changes in PMN phenotype after transepithelial migration, namely increased surface expression of CD66b and CD63, are due to an increase in the exocytosis of secondary and primary (NE-rich) granules [3,9]. In CF, a decrease in surface expression of CD16 of PMNs collected from sputum is also observed, which may lead to a decrease in phagocytic ability. In order to trap extracellular bacteria, PMNs can generate NETs, which are composed of chromatin complexed with granule effectors such as NE, cathepsin G, and myeloperoxidase [10]. In response to different stimuli involving bacteria, PMNs release pro-inflammatory cytokines or chemokines (e.g., IL-8) or lipid mediators (e.g., leukotriene B4 (LTB4)) that promote further blood PMN recruitment, thus creating an amplification loop that can impair tissue unless it is resolved [11,12].

In humans, PMNs are involved in many inflammatory complications, such as those linked to COPD, CF, severe asthma, cancer, or infection [13]. Glucocorticoids are commonly used to treat these diseases. However, glucocorticoids inhibit PMN cell death, which may promote further pathology. In general, PMNs often diverge from other leukocytes in their response to existing anti-inflammatory drugs [14,15]. Consequently, it is important to develop new candidate molecules able to decrease tissue recruitment and activation of PMNs in order to reduce potential tissue damage without inhibiting protective innate immune mechanisms dependent on PMNs.

In the last 25 years, phosphodiesterases (PDEs) have been identified as key targets in inflammatory diseases. PDEs are enzymes which hydrolyze cyclic adenosine monophosphate (cAMP) to AMP and cyclic guanosine monophosphate (cGMP) to GMP, both functioning as intracellular second messengers. PDE4 isoenzymes are expressed in inflammatory cells, including PMNs [16,17] wherein the three subtypes PDE4A, PDE4B, and PDE4D are found [18]. An example of drugs targeting at PDE4 currently in clinical use is roflumilast, a selective inhibitor mainly prescribed to patients with severe COPD [19], resulting in decreased airway inflammation and frequency of exacerbation [20]. However, side effects of roflumilast include diarrhea, pancreatitis, and weight loss, underlying the needs to develop alternatives [21].

We previously demonstrated that dihydropyridazinone-scaffold-based molecules reduce PDE4 activity and slightly decrease IL-8 release by LPS-stimulated blood PMNs in vitro [22]. Based on these preliminary results, we developed a new generation of potential anti-inflammatory agents. Two new pyridazinone-scaffold-based PDE4 inhibitors (compounds 4aa and 4ba, Figure S1) were synthesized and initially evaluated for their ability to regulate pro-inflammatory cytokine production by macrophages [23]. In the present study, we explore the potency of these compounds, compared to zardaverine (a commercially available drug from the same family), for their ability to: (1) modulate production of pro-inflammatory mediators and ROS as well as phagocytosis by stimulated human blood PMNs in vitro; (2) modulate degranulation by blood PMNs that have undergone transepithelial lung migration in vitro; and (3) limit tissue recruitment of blood PMNs in a murine air pouch model in vivo.

## 2. Results

### 2.1. Pyridazinone-Scaffold-Based Compounds Decrease the Release of Pro-Inflammatory Mediators by Activated PMNs

**Figure 1:**
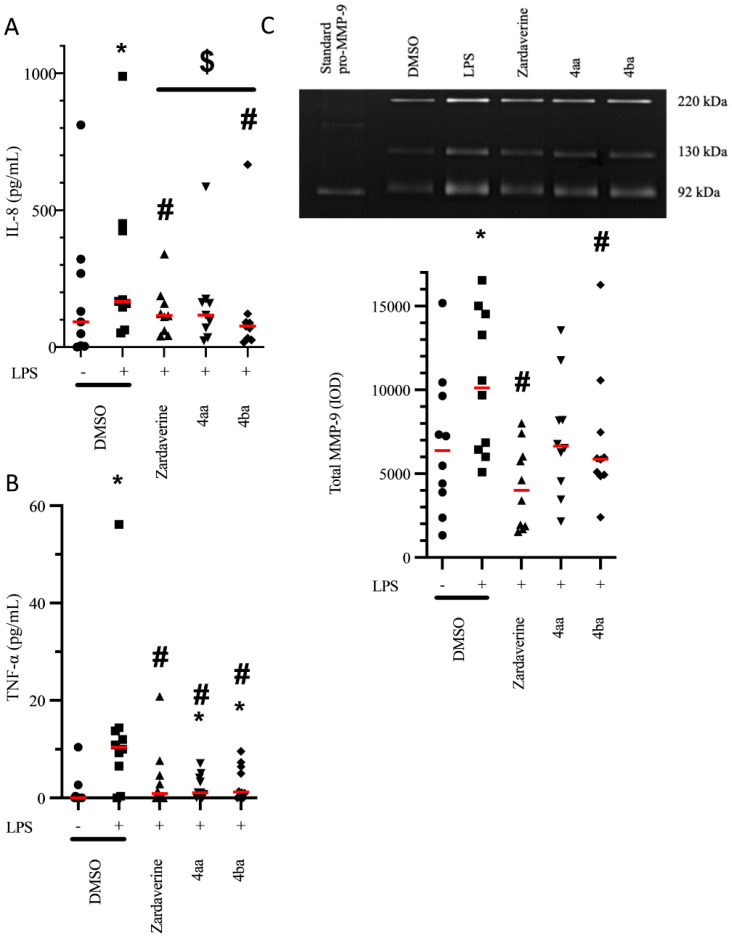
Pyridazinone-scaffold-based compounds decrease secretion of pro-inflammatory mediators. Released IL-8 (**A**) and TNF-α (**B**) quantified by ELISA and MMP-9 activity (**C**) quantified by gelatin zymography in cell culture supernatants after 4 h of treatment. DMSO (black circle), LPS + DMSO (black square), LPS + Zardaverine at 10 µM (black triangle), LPS + compound 4aa at 20 µM (inverted black triangle), LPS + compound 4ba at 20 µM (black diamond). *n* = 10, * *p* < 0.05 vs. DMSO, # *p* < 0.05 vs. LPS, $ *p* < 0.05 between indicated conditions.

To modulate inflammation, one approach consists of inhibiting recruitment and activation of blood leukocytes in tissue. Pro-inflammatory cells are recruited by chemokines such as IL-8, and activated by cytokines such as TNF-α. Having asserted the absence of toxicity of pyridazinone compounds (see supplementary materials and methods and Figure S2), we assessed their potential effect on the release of proinflammatory mediators by LPS-stimulated PMNs (4 h treatment). LPS stimulation increased IL-8 secretion by PMNs from 91.8 to 167.1 pg/mL (*p* = 0.004). This effect was blunted by treatment with zardaverine (115.1 pg/mL, *p* = 0.012) and compound 4ba (76.3 pg/mL, *p* = 0.004) (Figure 1A). Treatment with compound 4aa also trended toward decreasing IL-8 release (116.8 pg/mL), but this effect did not reach statistical significance (*p* = 0.102). Regarding TNF-α, LPS stimulation of PMNs increased secretion from 0 to 10.4 pg/mL (*p* = 0.004), and treatment with zardaverine, compounds 4aa and 4ba blunted this effect (0.9, 1.1, and 1.2 pg/mL, respectively, *p* < 0.05, Figure 1B).

After tissue recruitment and activation, PMNs may release proteases such as MMP-9 via degranulation, resulting in degradation of the extracellular matrix and furthering PMN recruitment. LPS stimulation of PMNs increased MMP-9-related gelatinolytic activity by 54% (*p* = 0.002) compared to that observed in non-stimulated PMNs. Treatment with zardaverine and compound 4ba induced a decrease in MMP-9-related gelatinolytic activity (by 60% and 33%, respectively, *p* < 0.05 for both, Figure 1C). Compound 4aa also trended toward decreasing MMP-9 activity by 31%, but this effect did not reach statistical significance (*p* = 0.082). 

**Figure 2:**
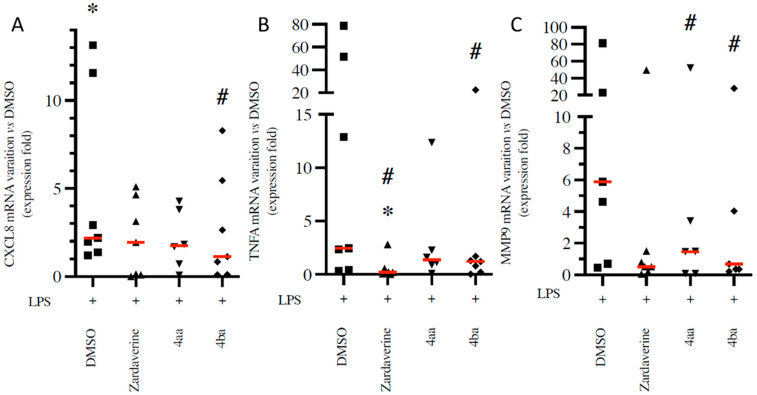
Pyridazinone-scaffold-based compounds decrease mRNA expression of pro-inflammatory mediators. CXCL8 (**A**), TNFA (**B**) and MMP9 (**C**) mRNA expression versus DMSO condition, quantified by qRT-PCR in cells after 4 h of treatment. LPS + DMSO (black square), LPS + Zardaverine at 10 µM (black triangle), LPS + compound 4aa at 20 µM (inverted black triangle), LPS + compound 4ba at 20 µM (black diamond). *n* = 7, * *p* < 0.05 vs. DMSO (value = 1), # *p* < 0.05 vs. LPS.

The effects of pyridazinone-scaffold-based compounds on pro-inflammatory mediator genes expression in LPS-stimulated PMNs was also evaluated. A LPS-induced increase in CXCL8 mRNA expression relative to DMSO condition (five-fold upregulation, *p* = 0.016) was decreased upon compound 4ba treatment by 46% (*p* = 0.031, Figure 2A). Zardaverine and compound 4ba decreased TNFA mRNA expression by 77% and 32% (*p* = 0.047 and *p* = 0.022, respectively) compared to LPS stimulation (Figure 2B). Finally, compound 4aa and 4ba decreased MMP-9 mRNA expression compared to LPS stimulation (35% and 50%, *p* = 0.047 and *p* = 0.031, respectively, Figure 2C).

Together, our data suggest that pyridazinone compounds, particularly compound 4ba, decrease the inflammatory release of IL-8, TNF-α and MMP-9 induced by LPS, and in turn, may inhibit PMN recruitment and activation, as well as resulting extracellular matrix degradation.

### 2.2. Pyridazinone-Scaffold-Based Compounds Do Not Impact Beneficial Functions in Activated PMNs

**Figure 3:**
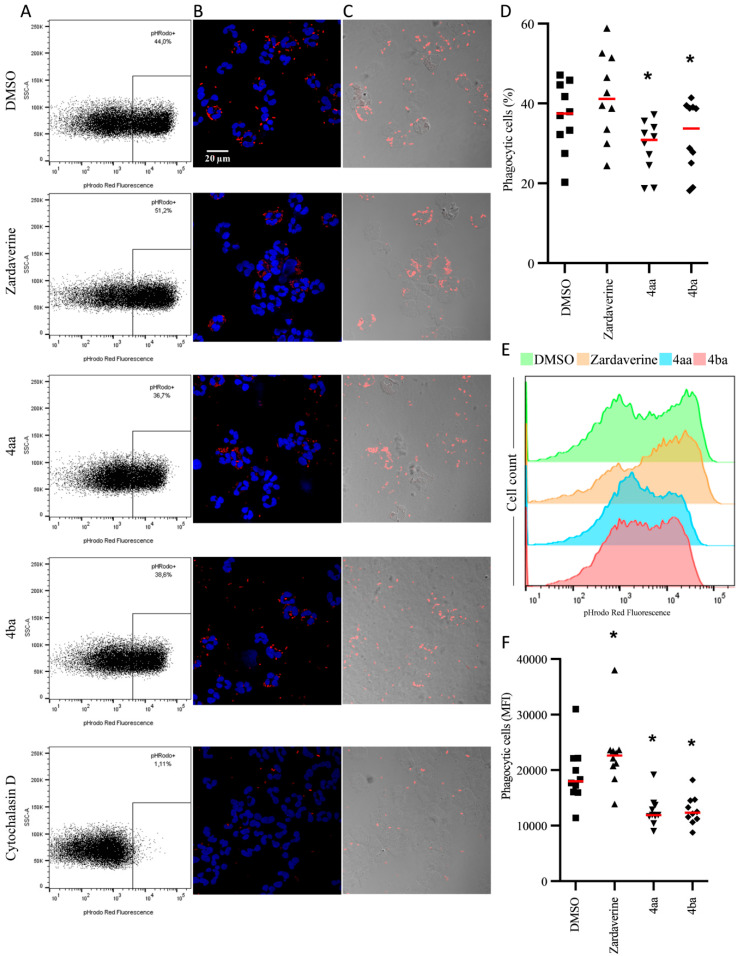
Pyridazinone-scaffold-based compounds decrease phagocytic abilities. Dot plot of PMNs exposed to pHrodo *E. coli* particles, and frequency of particle-positive cells (**A**). Representative fluorescent microscopy field of phagocytic cells (**B**) (nuclei (DAPI—blue) and pHrodo particles (red)) and transmitted-light microscopy (**C**) after 1 h of incubation with pHrodo *E. coli* particles and treatments. Cytochalasin D at 5 µg/mL was used at a negative control. Scale bar = 20 µm. Quantification of phagocytic cells (**D**). Representative histogram of MFI in phagocytes (**E**) and phagocytic capability (**F**) of PMNs after 1 h of incubation with pHrodo *E. coli* particles and treatments. DMSO (black circle), Zardaverine at 10 µM (black triangle), compound 4aa at 20 µM (inverted black triangle), compound 4ba at 20 µM (black diamond). *n* = 10, * *p* < 0.05 vs. DMSO.

**Figure 4:**
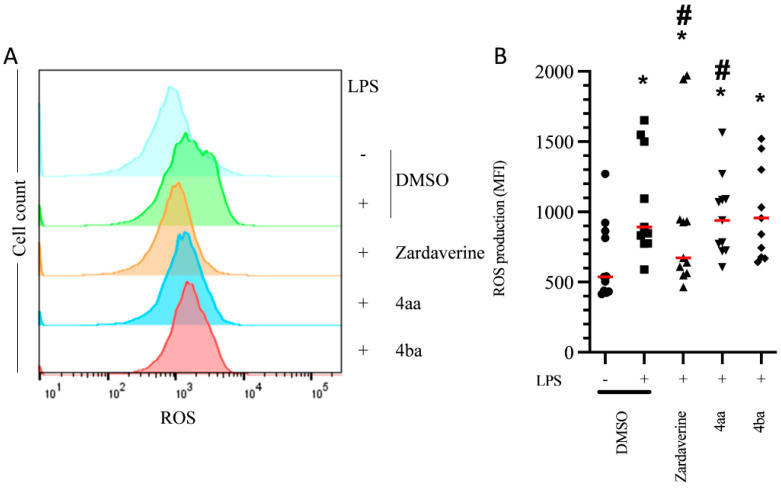
Pyridazinone-scaffold-based compounds slightly decrease ROS production in PMNs. Representative histogram (**A**) and quantification (**B**) of ROS production in cell culture after 4 h incubation with treatment. DMSO (black circle), LPS + DMSO (black square), LPS + Zardaverine at 10 µM (black triangle), LPS + compound 4aa at 20 µM (inverted black triangle), LPS + compound 4ba at 20 µM (black diamond). *n* = 11, * *p* < 0.05 vs. DMSO, # *p* < 0.05 vs. LPS.

Effective anti-inflammatory therapies should decrease deleterious impacts of inflammatory PMN activation without impacting beneficial functions such as bacteria killing. Here, we used pHrodo *E. coli* particles to evaluate the percentage of PMNs able to phagocyte in the presence or absence of pyridazinone compounds. Compounds 4aa and 4ba decreased the frequency of particle-positive PMNs (by 22% and 14%, respectively, *p* < 0.05) compared to the untreated DMSO condition (Figure 3A–D). To evaluate if pyridazinone compounds impact the quantity of particles phagocytosed by PMNs, we measured the MFI of the signal. Zardaverine increased MFI by 18% (*p* = 0.016), but compounds 4aa and 4ba decreased it by 44% and 43% (*p* = 0.002), respectively, compared to the untreated DMSO condition (Figure 3E,F). Once bacteria are in phagosomes, PMNs produce ROS that contribute to bacterial killing. While LPS stimulation increased ROS production by PMNs (66%, *p* = 0.001) compared to DMSO condition, this effect was blunted by zardaverine (25%, *p* = 0.025). Compounds 4aa and 4ba treatments induced a more modest reduction in ROS production (20% and 17%, *p* = 0.044 and *p* > 0.05, respectively) (Figure 4A,B). Thus, pyridazinone compounds do not fully inhibit PMN phagocytic ability and maintain their ROS production.

### 2.3. Pyridazinone-Scaffold-Based Compounds Modulate PMN Phenotype after Transepithelial Migration

**Figure 5:**
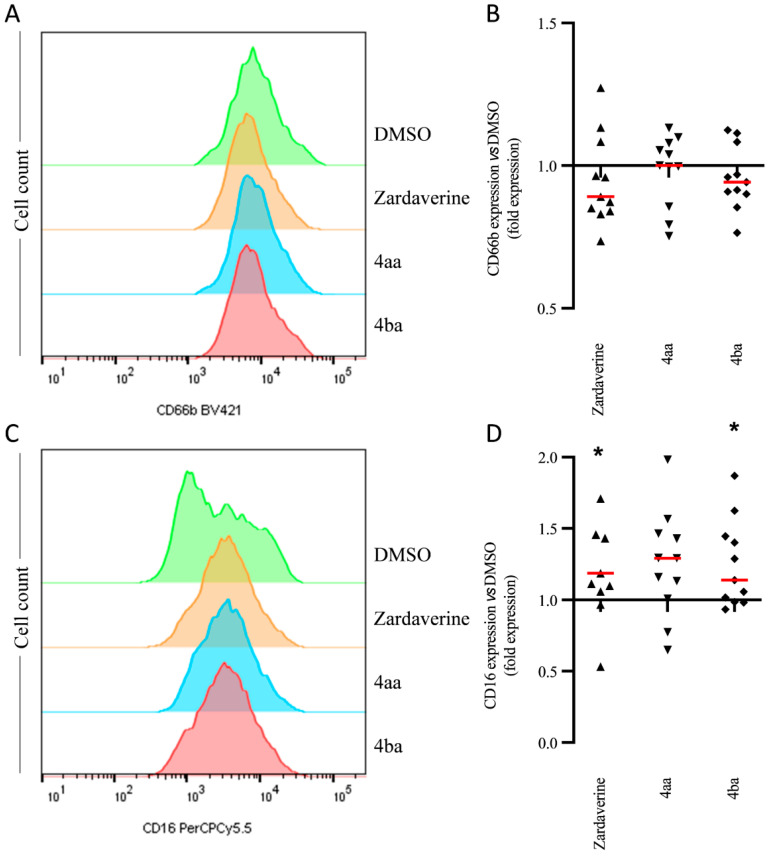
Pyridazinone-scaffold-based compounds modulate PMNs phenotype. Representative histogram and quantification of CD66b+ (**A**,**B**) and CD16+ (**C**,**D**) expression on PMN surface after 4 h of incubation with treatments. Zardaverine at 10 µM (black triangle), compound 4aa at 20 µM (inverted black triangle), compound 4ba at 20 µM (black diamond). *n* =11, * *p* < 0.05 vs. DMSO (value = 1).

Phenotypical changes in PMNs, through modulation of membrane receptor expression, have been demonstrated in activated PMNs that migrate through lamina propria-like structure and epithelial cell layers (Figure S3) [24]. We observed changes in CD66b and CD16 expression after PMN transmigration through an LPS-activated epithelium. All tested pyridazinone compounds failed to modulate CD66b expression (Figure 5A,B) on these transmigrated PMNs. However, zardaverine and compound 4ba increased CD16 expression by 30% (*p* = 0.049) and 20% (*p* = 0.027), respectively (Figure 5C,D). Thus, pyridazinone compounds partially restored PMN phenotype after transepithelial migration through increasing CD16 expression.

### 2.4. Pyridazinone-Scaffold-Based Compounds Decrease PMNs Recruitment In Vivo

**Figure 6:**
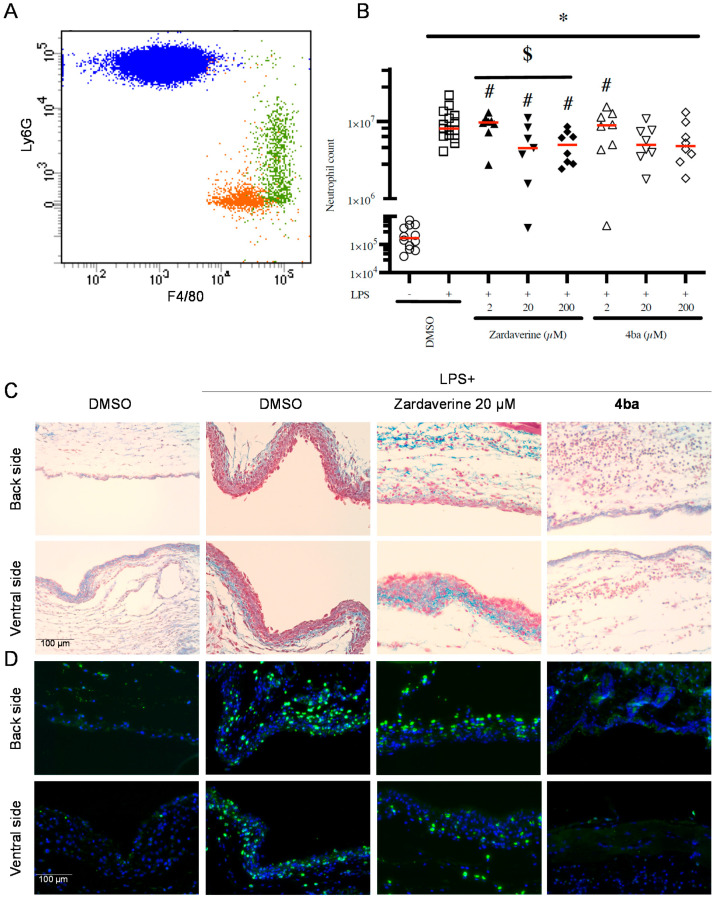
Pyridazinone-scaffold-based compounds decrease PMNs recruitment in vivo. Representative exudate population after LPS stimulation, monocytes (orange), macrophages (green) and PMNs (blue), (**A**). Quantification of PMNs (**B**) in exudate after 6 h of inflammation induction and treatment. DMSO (empty black circle), LPS + DMSO (empty black square), LPS + Zardaverine at 2 µM (full black triangle), 20 µM (full inverted black triangle), 200 µM (full black diamond), LPS + compound 4ba at 2 µM (empty black triangle), 20 µM (empty inverted black triangle), 200 µM (empty black diamond). Representative image of histology (**C**) and immunofluorescent staining (**D**) (nuclei in blue and Ly6G positive PMNs in green) of air pouch membrane, after treatment with DMSO, LPS + DMSO, LPS + Zardaverine at 20 µM and LPS + compound 4ba at 20 µM. *n* = 8 (zardaverine and 4ba conditions), *n* = 15 (DMSO and LPS conditions), * *p* < 0.05 vs. DMSO, # *p* < 0.05 vs. LPS, $ *p* < 0.05 between conditions.

Following up on our in vitro results, we tested the ability of zardaverine and compound 4ba to modulate leukocyte recruitment in vivo using the murine air pouch model of tissue inflammation. The total number of leukocytes in the exudate was counted, and PMNs were further gated and quantified by flow cytometry (Figure 6A). In this model, LPS increased recruitment of PMNs by 38-fold (*p* < 0.001) compared to DMSO condition (Figure 6B). The lowest concentration of pyridazinone compounds tested (2 µM) failed to counter PMN recruitment. At higher concentrations, zardaverine and compound 4ba decreased cell migration by up to 50% (*p* < 0.05). Flow cytometry data on exudates were in agreement with histological data showing reduced number of cells in the air pouch in the presence of pyridazinone compounds (Figure 6C). Moreover, we observed that among these cells, the PMN population was the most drastically reduced by 4ba treatment (Figure 6D). Thus, the pyridazinone compound 4ba successfully inhibits PMN recruitment to inflamed tissues in vivo.

## 3. Discussion

This study investigated the ability of pyridazinone-scaffold-based compounds to modulate LPS-induced recruitment and activation of PMNs in both in vitro and in vivo settings.

LPS is an essential component of the bacterial cell wall that induces human PMN activation, resulting in mRNA transcription and protein production and release affecting immune mediators such as IL-8 and TNF-α [25,26]. IL-8 is a chemoattractant for PMNs that facilitates their transmigration through the endothelium and epithelium [27,28]. Excessive recruitment of PMNs drives many inflammatory diseases such as psoriasis, arthritis, asthma, or COPD [29,30,31,32]. Roflumilast N-oxide, a PDE4 inhibitor, decreases IL-8 secretion by PMNs from healthy and COPD patients after LPS-induced inflammation [33]. Compared to our previously published results, the data presented here suggest that this new generation of pyridazinone compounds (notably, component 4ba) is threefold more effective than prior generations of pyridazinone compounds at similar concentrations, especially regarding inhibition of IL-8 release (55% reduction with 4ba compared to 15% with 3c compound in [22]). Efficient reduction of pro-inflammatory signaling is critical to limit tissue recruitment and activation of leukocytes. In addition to the here-presented effect on PMNs, it is noticeable that we previously evidenced such cytokine production modulation by monocytes/macrophages in vitro using compound 4ba, leading to the hypothesis of a global anti-inflammatory effect, not only on the granulocytes subset [23].

In physiological conditions, activated PMNs become apoptotic once their functions completed and are phagocytosed by macrophages, leading to resolution of inflammation [34]. TNF-α plays a key role in PMN apoptosis since it can induce apoptosis by binding to TNFR1 and subsequent activation of caspase-8. However, TNF-α also functions as a pro-inflammatory cytokine, facilitating PMN activation, adhesion, and increasing their ROS production [35,36]. One can speculate that anti-inflammatory benefits drawn from reduced TNF-α-mediated signaling would offset the potential dysfunction in apoptotic mechanisms and subsequent efferocytosis that may ensue. However, this remains to be elucidated.

Pulmonary emphysema as observed in COPD is due to elastin and collagen fiber disruption by proteases contained in PMN granules [37], as well as in extracellular vesicles [38]. NE, which is needed for pro-MMP9 activation, and MMP-9 release after PMN activation, can degrade basement membranes and facilitate PMN migration from blood [39,40]. As in previously published data [24], release of NE-rich primary granules, reflected by surface CD63 expression on PMNs, was not induced upon LPS stimulation in our experiments (Figure S3). In contrast, soluble MMP-9 was readily released by blood PMNs upon LPS stimulation. In prior studies, treatment with roflumilast reduced LPS-stimulated MMP-9 release by PMNs from both healthy subjects or patients with COPD [33]. Zardaverine, 4aa, and 4ba pyridazinone compounds also decreased soluble MMP-9 release upon LPS stimulation in our experiments. Whether pyridazinone compounds affect MMP-9 packaging in secreted extracellular vesicles remains to be determined. A decrease in MMP-9 release would tend to limit basement membrane disruption and cell migration during tissue inflammation. Interestingly, only the zymogen form of MMP-9 was detected in supernatants, which may mean that NE was not available to activate MMP-9, or that the autocatalytic mechanism of MMP-9 activation was dysfunctional [41]. Milara and coworkers showed that combined roflumilast treatment with dexamethasone decreased corticosteroid resistance of PMNs [33]. Further studies are needed to evaluate such a potential synergistic effect between corticosteroids and our newly synthesized pyridazinone compounds.

Phagocytosis is key to pathogen killing, starting with endocytosis and fusion of the phagosome with preformed granules containing antimicrobial compounds and enzymes [6]. Intracellular cAMP elevation decreases phagocytosis by inhibiting actin reorganization [42], which may underlie the effect of the PDE4 inhibitor rolipram on the phagocytic ability of PMNs [43]. Treatment with rolipram made PMNs less prone to bacterial uptake, reducing bacterial numbers per PMN but also reducing clearance of bacteria. Similar to rolipram, compounds 4aa and 4ba partially decreased PMN phagocytic ability, but kept the clearance activity. Antimicrobial compounds such as ROS are generated in activated PMNs and can be released intracellularly in the phagosome to kill bacteria [44]. ROS can also be released to kill bacteria extracellularly. On the other hand, excessive ROS release is involved in endothelium disruption and tissue injuries. Rolipram or roflumilast treatments have been shown to decrease ROS release by activated PMNs [45]. In our study, compounds 4aa and 4ba maintained normal ROS production by activated PMNs, whereas zardaverine decreased it. Thus, our newly synthesized pyridazinone-based molecules tended to preserve this important functional activity of PMNs. 

In pathological conditions, PMNs can be massively recruited to the airways. To better study this key process, Tirouvanziam and co-workers developed a lung epithelial transmigration model [24]. In CF, airway PMNs show increased surface expression of CD66b (reflecting secondary granule exocytosis) and CD63 (reflecting exocytosis of NE-rich primary granules), concomitant with decreased expression of CD16 [3]. Additionally, CF airway PMNs release many pro-inflammatory factors and lose their phagocytic capability. Butcher and colleagues have reported a correlation between CD16 expression and PMN phagocytic ability [46]. Because lung infections in COPD and CF disease are often due to Gram-negative bacteria [47,48], the ability of candidate drugs to restore PMN function after transmigration through LPS-exposed epithelium is of great interest. Our data showed that pyridazinone compounds do not impact surface expression of CD63 or CD66b but increase that of CD16. Further studies are needed to determine whether this may result in a restoration of PMN phagocytic abilities upon recruitment to infected tissues.

To approach the effects of anti-inflammatory drugs in situ, especially in acute inflammation, a 6-day-old murine air pouch model was developed [49,50]. As recovering exudate and performing histology of the air pouch membrane is easy in this model, we used this approach to evaluate the effects of a pyridazinone-scaffold-based compound on inflammatory cell recruitment in vivo. Consistently with prior data on other PDE4 inhibitors (cilomilast and roflumilast) in other models [51,52] as well as evidenced recently in monocytes/macrophages [23], pyridazinone-scaffold-based compounds reduced LPS-induced PMN recruitment both in exudate and in tissue within this model. Precise mechanisms involved in reducing PMN migration or retention upon treatment with our pyridazinone-scaffold-based compounds remain to be elucidated. One plausible mechanism involves inhibition of F-actin formation and chemoattractant-mediated shape changes, as previously demonstrated upon roflumilast treatment in fMLP- and LPS-stimulated cells [53,54]. Such modifications in cellular behavior after PDE4 inhibition were attributed to the cAMP/epac1 (exchange protein directly activated by cAMP 1) pathway [55]. Another plausible mechanism involves inhibition of Src-family kinases by PKA activation, which was shown to disrupt P-selectin-mediated firm adhesion of PMNs on platelets upon rolipram treatment [56].

## 4. Materials and Methods

### 4.1. Media and Reagents

Power SYBR^®^green mastermix and cDNA reverse transcription kit were purchased from Applied Biosystems (Villebon-sur-Yvette, France). Human anti-CD63-PE (clone H5C6), human anti-CD16-BV421 (clone G10F5), human anti-CD66B-PerCP-Cy™5.5 (clone 3G8), human Fc Block, murine Fc block, murine anti-Ly6G-PE (clone 1A8), murine anti-F4/80-BV421 (clone T45-2342), fixable viability stain 510 (FVS510), and lyse/fix Buffer 5X were purchased from BD Biosciences (Le Pont de Claix, France). Recombinant rabbit anti-mouse Ly6G (EPR22909-135) was from Abcam (Cambridge, UK). Goat anti-rabbit IgG biotinylated antibody and Bloxall^®^ Endogenous Blocking Solution were purchased from Vector Laboratories (Burlingame, CA, USA). Fluorescent Mounting Medium^®^ was from Agilent-DAKO (Santa Clara, CA, USA). Ethylene diamine tetracetic acid (EDTA), Tris(hydroxymethyl)aminomethane (TRIS), Triton-X100, sodium dodecyl sulfate (SDS), and Coomassie Blue were purchased from Bio-Rad (Marnes-la-Coquette, France). Dulbecco’s minimal essential medium (DMEM)/Ham’s F-12 50/50 mix was purchased from Corning (Boulogne-Billancourt, France). Zardaverine was purchased from Enzo Life Sciences (Villeurbanne, France). MasterPure™ RNA Purification Kit was purchased from Epicentre^®^ Biotechnologies (Euromedex, Souffelweyersheim, France). Muse^®^ Oxidative Stress Kit, fuchsin and hematoxylin were purchased from Merck Millipore (Molsheim, France). Ultroser™ G was purchased from PALL (Saint-Germain-en-Laye, France). Polymorphprep™ was purchased from ProteoGenix (Schiltigheim, France). Penicillin-streptomycin (Pen-Strep, 10,000 U.mL^−1^), dimethylsulfoxide (DMSO), lipopolysaccharide (LPS) from *E. coli* 0111:B4, collagen type I solution from rat tail, calcium chloride dihydrate, gelatin from bovine skin, acrylamide/bis-acrylamide 30% solution, recombinant human pro-MMP9, cytochalasin D and aniline blue were purchased from Sigma Aldrich (Saint-Quentin Fallavier, France). Elisa DuoSet kits for human IL-8 and TNF-α were from purchased R&D systems (BioTechne, Rennes, France). RPMI 1640 medium, DPBS, DAPI, AlexaFluor^®^488-conjugated Streptavidin, Paraffin Shandon™ and pHrodo™ Red *E. coli* BioParticles™ Conjugate for Phagocytosis were from ThermoFisher Scientific (Illkirch-Graffenstaden, France). Sodium chloride, acetic acid, methanol, and formaldehyde 4% were purchased from VWR (Strasbourg, France). IsoFlo^®^ was purchased from Zoetis (Malakoff, France).

### 4.2. Compound Preparation

Pyridazinone derivatives bearing indole (4aa) or 5-methoxy-indole (4ba) moieties were obtained by a three-step synthesis starting from levulinic acid. After preparation of the α, β-unsaturated levulinate intermediate [57] regiospecific introduction of the heterocyclic system was carried out via a Friedel–Crafts-type reaction, followed by condensation with hydrazine leading to the formation of the desired functionalized dihydropyridazinones with good yields [58,59,60]. A final oxidation step using manganese dioxide allowed us to obtain corresponding pyridazinone derivatives named 4aa and 4ba. For biological experiments, zardaverine (commercially available PDE3/PDE4 inhibitor), as well as compounds 4aa and 4ba were solubilized in DMSO. Stock solution concentration was adjusted to reach a 0.5% DMSO final concentration in contact with cells.

### 4.3. Collection of Blood Samples, Cell Isolation and Culture

Blood samples were provided from “Etablissement Français du Sang Grand Est” (Authorization ALC/PIL/DIR/AJR/FO/606 Reims, France) following ethical legal regulation (Article R1243-57), in accordance with our authorization and registration number DC-2014-2262 given by the French Ministry of Research. Blood was collected in EDTA tubes (BD Vacutainer^®^ K2E) and PMNs were purified using Polymorphprep^TM^ according to the manufacturer’s protocol. Contaminating red blood cells were removed via hypotonic shock. Purified PMNs were resuspended in complete medium (RPMI 1640 Glutamax with 2.5% heat-inactivated autologous human serum and 1% Pen-Strep) and represented more than 93% of the cells (see supplementary materials and methods and Figure S4). PMNs were at least 97% viable as estimated by the trypan blue exclusion method. PMNs were cultured for 4 h in 24-well plates with 1 mL of complete medium at 37 °C in a humidified atmosphere with 5% CO_2_ at 1 million cells per well. Inflammatory conditioning was obtained by addition of LPS at 10 ng/mL for 4 h [22]. In all conditions, PMNs were cultured in 0.5% DMSO adjusted medium. Based on preliminary setup experiments (Figure S5), treatments were performed with pyridazinone-scaffold-based molecules at 20 µM for compounds 4aa and 4ba and 10 µM for zardaverine for 4 h. For all experiments, pyridazinone-scaffold-based molecules were first provided to the cells, immediately followed by LPS treatment to avoid priming effect of the cells. After incubation, cell suspensions were spun at 600 *g* for 10 min to collect both supernatants and cell pellets.

### 4.4. ELISA

IL-8 and TNF-α concentrations in PMN-conditioned supernatants were measured by ELISA DuoSet kits following the manufacturer’s instructions. Controls included non-stimulated cells and medium alone. Level of IL-8 and TNF-α were calculated per a human recombinant protein standard curve. All measurements were performed at 450 nm corrected at 570 nm on a BMG Labtech Fluostar Optima^®^ (ThermoFisher Scientific, Illkirch-Graffenstaden, France).

### 4.5. Gelatin Zymography

Gelatin zymography was used to detect pro-enzyme and active forms of soluble MMP-9 in PMN-conditioned supernatants. Samples were diluted in RPMI to deposit 1 µg of total protein per lane. First, 10% acrylamide separation gel containing 0.1% of porcine gelatin in TRIS buffer (pH 8.8, 1.5 M) was produced and then surmounted by a 4% acrylamide compression gel in TRIS buffer (pH 6.8, 0.5 M). The migration was carried out in denaturing conditions (0.1% SDS in the gel) for 30 min at 90 V in the compression gel, and then at 180 V in the separation gel using a Bio-Rad Power Pac 200. After migration, gels were washed in 1.25 g/L Triton X-100 solution, 2 times for 30 min, then incubated for 18 h at 37 °C in incubation buffer at pH 7.6 (TRIS 30 g/L, sodium chloride 58.5 g/L and calcium chloride 3.7 g/L). Gels were then stained for 30 min with Coomassie blue G-250 1 g/L, 100 mL/L acetic acid and 400 mL/L methanol in water and destained with acetic acid 100 mL/L and methanol 200 mL/L in water (2 × 30 min). Gel digitalization was performed on a Vilber Lourmat CN-UV/WL system controlled by the BioCapt^®^ software. Gelatin degradation bands were semi-quantified using the ImageJ Gel Analyzer module. Three forms of MMP-9 were detected in PMNs: pro-MMP-9 (92 kDa), pro-MMP9 complexed with lipocalin (130 kDa also called MMP-9/NGAL complex) and pro-MMP9 homodimers (220 kDa) [61,62].

### 4.6. RNA Purification and Reverse-Transcription

Total RNAs were extracted and cleaned up from cell pellets with MasterPureTM RNA Purification Kit in accordance with the manufacturer protocol. RNA purity was assessed by measuring the absorbance ratio at 260/280 nm (NanoDrop 2000c, ThermoFisher Scientific, Illkirch-Graffenstaden, France), which ranged between 1.8 and 2. Total RNAs (250 ng) were reverse transcribed into cDNA using a high-capacity cDNA reverse transcription kit following the manufacturer’s instructions (Eppendorf^®^ Mastercycler, ThermoFisher Scientific, Illkirch-Graffenstaden, France). Primers were designed for MMP9, CXCL8, TNF and RPS18 genes and their efficiencies were determined (Table 1). Transcription products were amplified by qRT-PCR on an Applied Biosystems^TM^ StepOnePlusTM Real-Time PCR System (ThermoFisher Scientific, Illkirch-Graffenstaden, France). After a first denaturation step at 95 °C for 10 min, qRT-PCR reactions were performed according to a thermal profile that corresponds to 40 cycles of denaturation at 95 °C for 15 s, annealing and extension at 60 °C for 1 min. Data collection was performed at the end of each annealing/extension step. The third step, which consists of a dissociation process, is performed to ensure the specificity of the amplicons by measuring their melting temperature (Tm). Data analysis was performed with the Applied Biosystems^TM^ StepOne^TM^ Software v2.3.

### 4.7. Phagocytosis

PMN phagocytic abilities were evaluated using pHrodo™ Red *E. coli* BioParticles™ Conjugate. Two hundred and fifty thousand PMNs were incubated in complete medium with particles at 2.5 µg/mL for 1 h. Negative control samples were composed of cells with particles and the actin polymerization inhibitor cytochalasin D at 5 µg/mL. PMNs were immediately used for flow cytometry acquisition with a BD LSRFortessa™ system (BD Biosciences, San Jose, CA, USA). pHrodo™ Red *E. coli* BioParticles™ Conjugate was excited with the 561 nm excitation laser. The 585/15 nm emission filter was used to detect the resulting fluorescence signal. Twenty-thousand cells were collected per sample. Cells were first selected by a FSC-A/SSC-A gate excluding dead cells and subcellular debris, and single cells were then selected by a SSC-H/SSC-W gate. Data analysis was performed with FlowJo software (BD Biosciences). The events within this region were depicted in a pHrodo™ Red fluorescence histogram. Results report cells with pHrodo™ signal measured as median fluorescence intensity (MFI).

Additionally, one hundred microliter suspension of cells and particles was deposited on a glass slide using Cytocentrifuge Cytospin™ (Thermo Scientific, Illkirch-Graffenstaden, France) for 8 min at 600 *g*. Cells were then fixed in 4% paraformaldehyde at 37 °C for 15 min. After fixation, cells were stained with DAPI (at 1 µg/mL in distilled water for 5 min). Coverslips were mounted on glass slide using one drop of Fluorescent Mounting Medium^®^ after an ultimate rinse in PBS. After drying overnight at room temperature, observations and acquisitions were performed using an inverted confocal microscope LSM 710 NLO Zeiss. Objective with a 63-fold magnification.

### 4.8. Oxidative Stress Quantification

ROS were quantified using the Muse^®^ Oxidative Stress Kit. Following the manufacturer’s instructions, cells were spun before incubation with the working solution. After 30 min, cells were immediately used for flow cytometry acquisition with a BD LSRFortessa™ system. Dihydroethidium (DHE) was excited with the 488 nm excitation laser. The 585/15 nm emission filter was used to detect the resulting fluorescence signal. Ten thousand cells were collected per sample and analyzed as described above.

### 4.9. Transepithelial Migration

As previously described [24], Alvetex scaffold (Reprocell, Glasgow, United Kingdom) were activated with 70% ethanol, coated with rat-tail collagen I (3 mg/mL) and seeded with epithelial cell line H441 (ATCC^®^ HTB-174TM). Growth and differentiation of cells was performed at the air–liquid interface (ALI) using DMEM/F12 with 10% Ultroser G for 2 weeks to obtain a fully differentiated epithelium. For transmigration (TM) experiments, H441-ALI scaffolds were flipped and the apical compartment (facing down) was exposed to LPS at 10 ng/mL. For each well, one million PMNs were put on the basal compartment (facing up) and allowed to migrate for 14 h. The PMNs that transmigrated were then collected, pooled and spun at 600 *g* for 10 min before being transferred into new wells to get in contact with drugs. Two hundred and fifty thousand PMNs were incubated for 4 h with pyridazinone-scaffold-based compounds in serum and LPS free RPMI medium. Finally, PMNs were collected and stained for flow cytometry with FVS510, and the following fluorescent antibodies: anti-CD16, anti-CD66b and anti-CD63. After PBS-EDTA (2.5 mM) washes, cells were fixed and used for flow cytometry acquisition with a BD LSRFortessa™ system (BD Biosciences, San Jose, CA, USA). Anti-CD66b-BV421 and FVS510 were excited with the 405 nm excitation laser and detected with the emission filters 450/50 nm and 525/50 nm, respectively. Anti-CD16-PerCPCy5.5 was excited with the 488 nm excitation laser and detected with the emission filter 710/50 nm. Anti-CD63-PE was excited with the 561 nm excitation laser and detected with the emission filter 585/15 nm. Ten thousand cells were collected per sample. At the first stage, single cells were selected by a FSC-H/FSC-A gate. On this population, cells were selected by a FSC-A/SSC-A gate excluding subcellular debris. Then, live PMNs were selected by a CD66b+/FV510- gate. The events within this region were depicted in three histograms showing MFIs for CD66b-BV421, CD16-PerCPCy5.5 and CD63-PE. Data analysis was performed with FlowJo software. Changes in cell phenotype after TM were validated using staining with FVS510 (>90% viability), anti-CD16, anti-CD66b and anti-CD63 (Figure S3).

### 4.10. Air Pouch Model of PMN-Driven Tissue Inflammation

Animal experiments were performed on mice housed in a controlled environment (temperature: 21 ± 2 °C, relative humidity: 65 ± 15%, natural alternating light cycle/darkness, health university campus, Reims, France, agreement n°B514543) in accordance with protocols approved by the Regional Ethics Committee on Animal Experimentation (CEEA n°056) and the Ministry of Agriculture, under the direction of investigators certified for animal experiments following protocol APAFIS#13375-2017121218136235v9. Seven-week-old BALB/cJRj mice were acclimated for 1 week before air pouch induction. Before each injection, mice were anesthetized using IsoFlo. At D0, three milliliters of 0.2 µm filtered air were injected with 10 mL syringe and 27G needle, subcutaneously, in the middle of the mice’s back. At day 3, injection was repeated. At D7, 2 mL was injected: 1 mL of DMSO, zardaverine or compound 4ba (two times concentrated for final concentration 0.5% for DMSO and 2, 20, or 200 µM for pyridazinone-scaffold-based compounds), immediately followed by injection of 1 mL of LPS (two times concentrated for final concentration 10 ng/mL) to induce inflammation. Control mice were injected with 2 mL of 0.5% DMSO (negative control) or LPS 10 ng/mL in 0.5% DMSO (positive control). After 6 h, intra-cardiac blood was collected under gaseous anesthesia before sacrifice of the animals. To remove the exudate from the air pouch, 2 mL of PBS-EDTA at 5 mM was injected, and after a 30 s soft massage of the pouch, exudate was collected. Cells from the exudate were washed and counted using a Kova^®^ slide (Dutsher, Brumath, France). To evaluate leukocyte subsets and discriminate monocytes and macrophages, cells were stained with fluorescent antibodies anti-Ly6G and anti-F4/80. After a wash, cells were fixed (BD LyseFix^TM^) and used for flow cytometry acquisition with a BD LSRFortessa™ system. F4/80-BV421 was excited with the 405 nm laser and detected with the emission filter 450/50 nm. Ly6G-PE was excited with the 561 nm laser and detected with the emission filter 585/15 nm. One hundred thousand cells were collected per sample. At the first stage, single cells were selected by a FSC-H/FSC-A gate. On this population, cells were selected by a FSC-A/SSC-A gate, excluding subcellular debris. Then, PMNs were selected by a Ly6G-PE + gate on a SSC-A/Ly6G-PE dot plot. Macrophages and monocytes were discriminated on a SSC-A/F4/80-BV421 dot plot. Data analysis was performed with FlowJo software.

For histological analysis, collected air pouches were fixed in formaldehyde 4%, washed and dehydrated by gradually replacing water in the sample with alcohol, then alcohol was replaced by xylene. After samples were embedded in paraffin, multiple serial sections were taken at 3.5 µm thickness. Sections were rehydrated and stained with aniline blue, hematoxylin and fuchsin. To identify PMNs, immunofluorescent staining was performed on the following sections. They were blocked with Bloxall^®^ Endogenous Blocking Solution and incubated overnight at 4 °C with the primary anti-Ly6G antibody (1:4000 in 1% BSA). Then, sections were incubated for 30 min at room temperature with secondary goat anti-rabbit antibody (1:50 in 1% BSA). Controls were carried out by omission of the primary antibodies. Sections were counterstained with DAPI (1:3000) to visualize the cell nuclei. Sections were then mounted thanks to Dako Fluorescent Mounting Medium^®^ with a coverslip and observations and acquisitions were performed using a Zeiss Axiovert 200 M inverted microscope and coupled AxioVisionTM v2.8 software.

### 4.11. Statistics

Each experiment was performed on cells from 7–11 independent human donors or 8–15 mice. The significance of the biological results was assessed with a non-parametric approach owing to a lack of normal distribution of the assessed variables. A Kruskal–Wallis test followed by a post hoc exact and stratified (when appropriate) Wilcoxon Mann–Whitney test (StatXact 7.0, Cytel Inc., Cambridge, MD, USA) were used. A value of *p* ≤ 0.05 was chosen as statistically significant. In all figures, red bars represent median values, limits of the boxes represent the first and third quartile, and bars represent the first and ninth decile. Black dots represent average values when relevant.

## 5. Conclusions

In conclusion, this study provides proof-of-concept data suggesting therapeutic potential for the new generation of PDE4-inhibitory pyridazinone compounds tested here. Although these pyridazinone-scaffold-based compounds warrant further investigation to determine their exact mechanism of action and efficacy in more complex in vivo models, they hold promise as novel therapeutic options for the treatment of acute inflammatory diseases induced by PMNs, owing to their ability to reduce the release of inflammatory mediators and subsequent recruitment of PMNs without impacting their bactericidal functions.

## Figures and Tables

**Table 1:** Nucleotide sequences of primers used for qRT-PCR.

Target Gene	Sequences		Efficiency
	Forward Primer (5′⟶3′)	Reverse Primer (5′⟶3′)	
MMP9	GAACCAATCTCACCGACAGG	GCCACCCGAGTGTAACCATA	1.94
CXCL8	AGACAGCAGAGCACACAAGC	CTCCTTGGCAAAACTGCAC	1.94
TNF	CAGCCTCTTCTCCTTCCTGAT	GCCAGAGGGCTGATTAGAGA	1.95
RPS18	TGCGAGTACTCAACACCAACA	GCATATCTTCGGCCCACA	1.96

## Data Availability

The data presented in this study are available on request from the corresponding author.

## Supplementary Materials

The following supporting information can be downloaded at: https://www.mdpi.com/article/10.3390/ijms23137226/s1.
