# Supplementary material for: Inhibition of Recruitment and Activation of Neutrophils by Pyridazinone-Scaffold-Based Compounds
Source: Int J Mol Sci. 2022 Jun 29;23(13):7226. doi: 10.3390/ijms23137226 (PMC9266889; doi:10.3390/ijms23137226)
Supplement: Supplementary file 1 [file ijms-23-07226-s001.zip › Template-IJMS Supp Info_R1.pdf]

## Supplementary Material and Methods

*PMN staining for flow-cytometry analysis*

After PMN isolation, cells were stained using anti-human CD66b-BV421 and FVS510 (BD Biosciences, Le Pont de Claix, France). After wash, cells were fixed and used for flow cytometry acquisition with a BD LSRFortessa™ system (BD Biosciences, San Jose, CA). CD66b-BV421 and FVS510 were excited with the 405 nm laser and detected with emission filters 450/50 nm and 525/50 nm, respectively. At the first stage, single cells were selected by a FSC-H/FSC-A gate. On this population, cells were selected by a FSC-A/SSC-A gate excluding subcellular debris and FVS510+ gate excluding dead cells. Ten thousand cells were collected per sample. Data analysis was performed with FlowJo software (Tree Star, Stanford University, USA).

*Lactate dehydrogenase quantification*

Cytotoxicity was determined by measuring lactate dehydrogenase activity in cell culture supernatant according to the manufacturer's protocol (Roche, Sigma Aldrich, Saint-Quentin Fallavier, France). The absorbance was read at 492 nm corrected at 570 nm on a BMG Labtech Fluostar Optima®.

## Supplementary Figure Legends

**Figure S1.** Chemical structure of compounds 4aa and 4ba.
**Figure S2.** Lactate dehydrogenase activity in PMN supernatants after 4h of treatment. DMSO (black circle), LPS + DMSO (black square), LPS + Zardaverine at 10 µM (black triangle), LPS + compound 4aa at 20 µM (inverted black triangle), LPS + compound 4ba at 20 µM (black diamond). n=7.
**Figure S3.** Representative histogram and quantification of initial shifts in CD66b (A), CD63 (B) and CD16 (C) expression for PMNs before (black stars) and after (grey stars) transmigration. n =10.
**Figure S4.** Representative dot plot of purified blood cells after Polymorphprep™ (left panel) and histogram of CD66b+ cells (right panel).
**Figure S5.** Pyridazinone scaffold-based compounds concentration dependent-effect on IL-8 secretion quantified by ELISA after 4h of treatment: Zardaverine (A), 4aa (B) and 4ba (C). DMSO (black circle), LPS + DMSO (black square), LPS + Zardaverine at 10 µM (black triangle), LPS + compound 4aa at 20 µM (inverted black triangle), LPS + compound 4ba at 20 µM (black diamond). n=9, * $p < 0.05$ *vs* DMSO, # $p < 0.05$ *vs* LPS.
