# Supplementary figures and images for: Inhibition of Recruitment and Activation of Neutrophils by Pyridazinone-Scaffold-Based Compounds
Source: Int J Mol Sci. 2022 Jun 29;23(13):7226. doi: 10.3390/ijms23137226 (PMC9266889; doi:10.3390/ijms23137226)

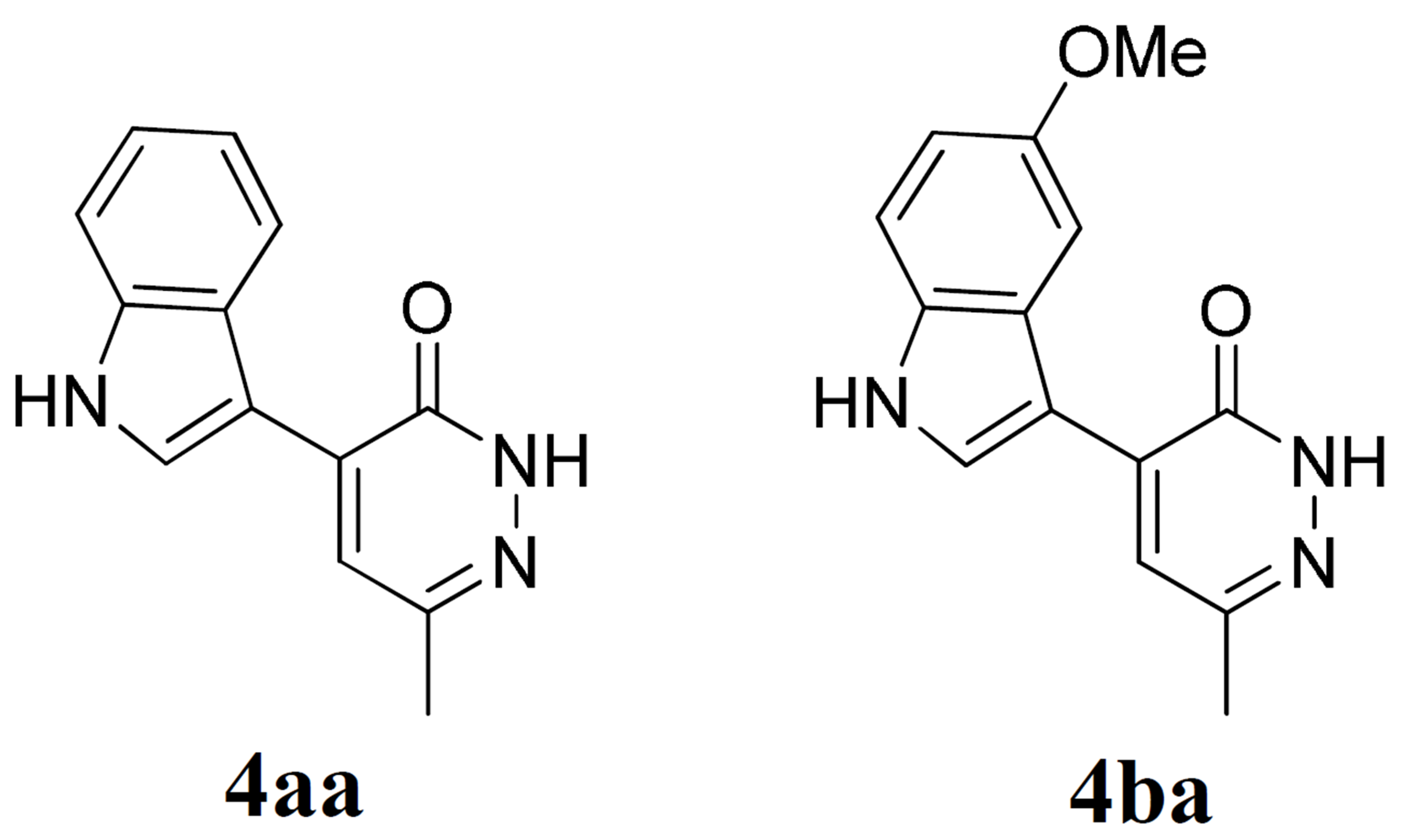

Supplement: Supplementary file 1 [file ijms-23-07226-s001.zip › Figure S1.tif]

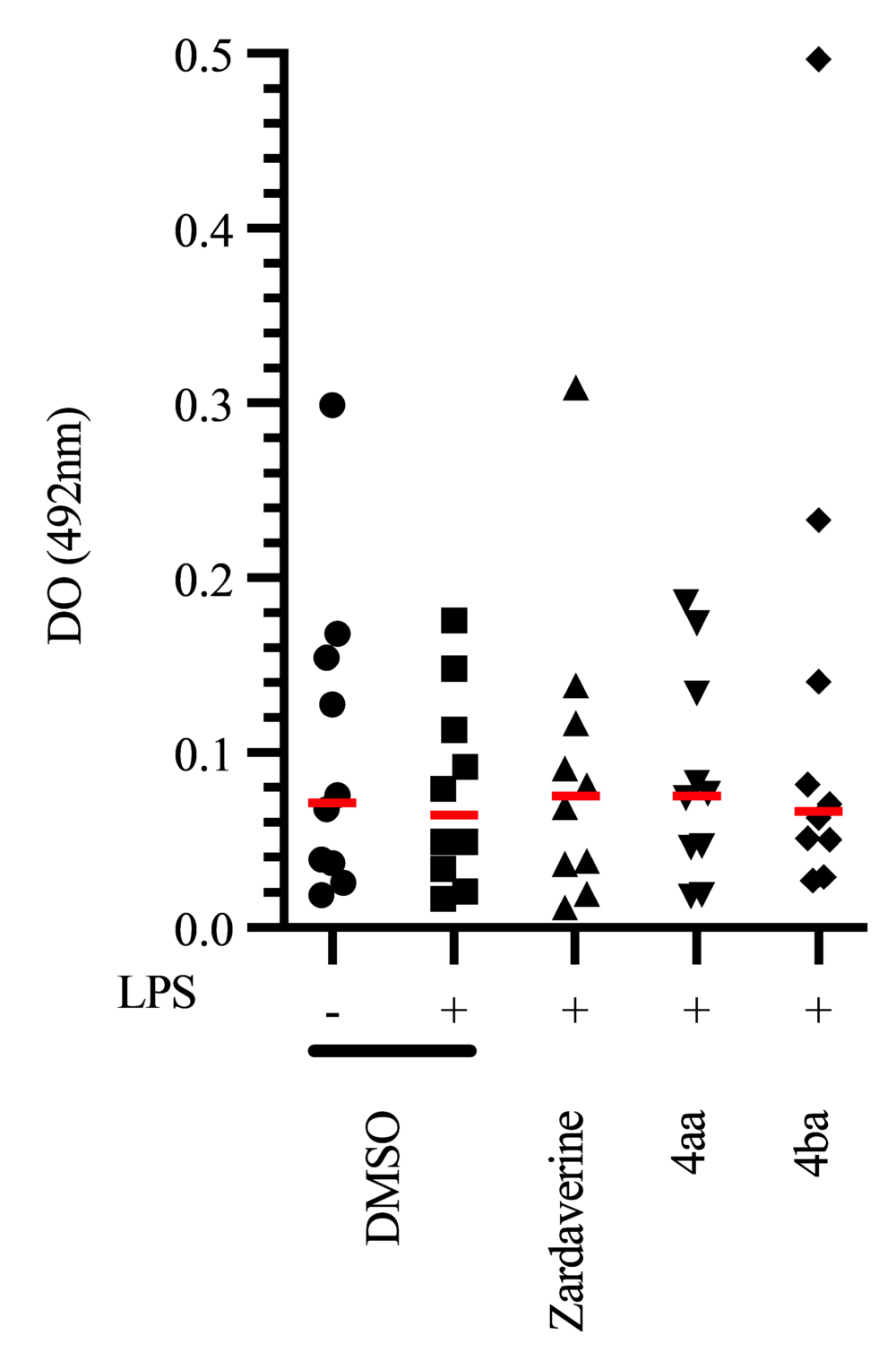

Supplement: Supplementary file 1 [file ijms-23-07226-s001.zip › Figure S2.tif]

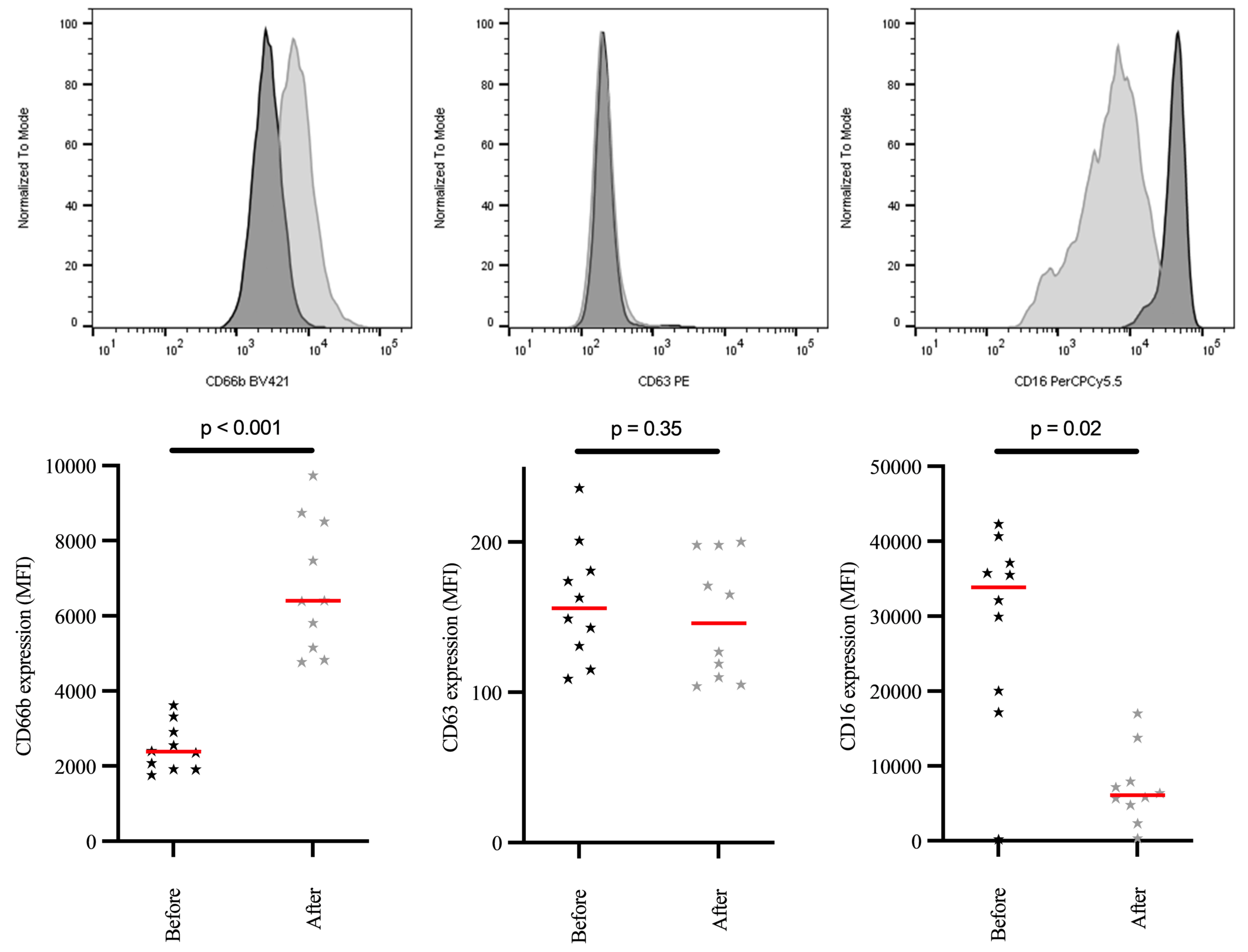

Supplement: Supplementary file 1 [file ijms-23-07226-s001.zip › Figure S3.tif]

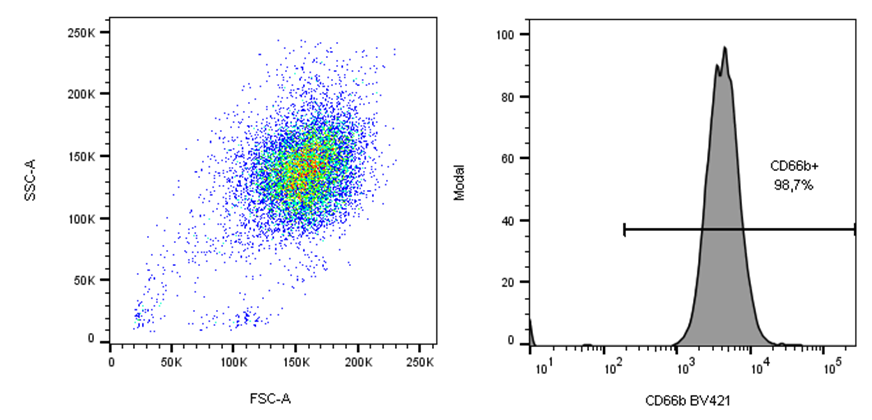

Supplement: Supplementary file 1 [file ijms-23-07226-s001.zip › Figure S4.tif]

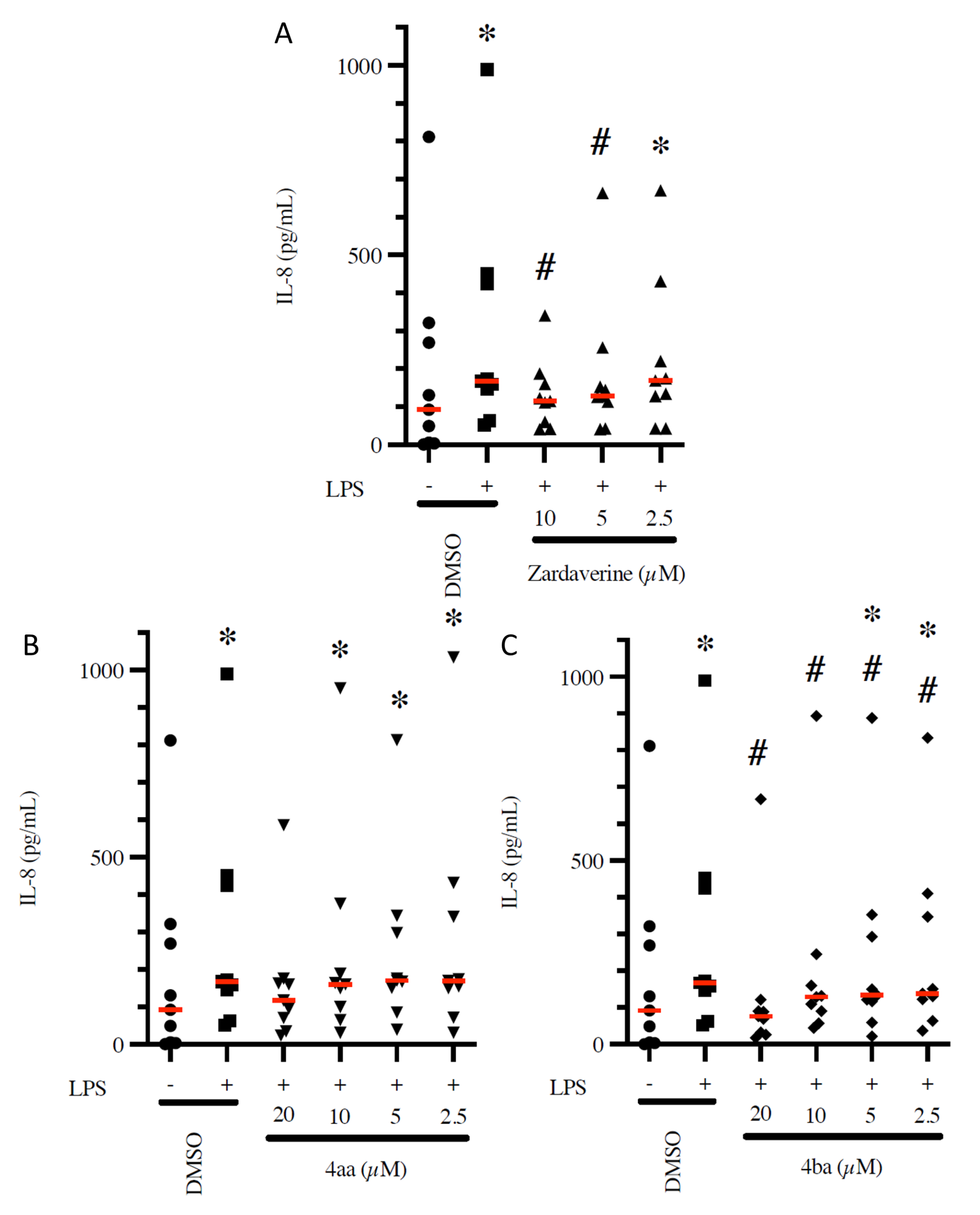

Supplement: Supplementary file 1 [file ijms-23-07226-s001.zip › Figure S5.tif]
